# Blocking Type I Interferon Signaling Rescues Lymphocytes from Oxidative Stress, Exhaustion, and Apoptosis in a Streptozotocin-Induced Mouse Model of Type I Diabetes

**DOI:** 10.1155/2013/148725

**Published:** 2013-03-07

**Authors:** Hany M. Ibrahim, Ibrahim A. El-Elaimy, Heba M. Saad Eldien, Badr Mohamed Badr, Danny M. Rabah, Gamal Badr

**Affiliations:** ^1^Zoology Department, Faculty of Science, Minufiya University, Shebin El-Kom, Egypt; ^2^Histology Department, Faculty of Medicine, Assiut University, Assiut, Egypt; ^3^Department of Urology/Surgery, College of Medicine, King Saud University, Saudi Arabia; ^4^Princess Al-Johara Al-Ibrahim Center for Cancer Research, College of Medicine, King Saud University, P.O. Box 7805, Riyadh 11472, Saudi Arabia; ^5^Zoology Department, Faculty of Science, Assiut University, Assiut 71516, Egypt

## Abstract

Elevated levels of type I interferon (IFN) during type 1 diabetes mellitus (T1D) are associated with a defective immune response. In the present study, we investigated whether blocking type I IFN signaling during streptozotocin- (STZ-) induced T1D in mice improves lymphocyte proliferation and escape from continuous apoptosis. Three groups of mice were examined: diabetic mice, type I IFN signaling-incompetent diabetic mice, and control nondiabetic mice. We first found that diabetes induction was accompanied by an elevation in the plasma levels of reactive oxygen species (ROS), hydroperoxide, malondialdehyde (MDN), and the proinflammatory cytokines IL-1**α**, IL-1**β**, IL-6, and CXCL10. Blocking type 1 IFN signaling in diabetic mice significantly decreased the levels of oxidative stress and proinflammatory cytokines. In addition, lymphocytes from diabetic mice exhibited a marked reduction in their proliferative capacity, increased apoptosis, upregulation of the exhaustion marker PD-1, and aberrant phosphorylation of STAT1, STAT2, AKT and I**κ**B-**α**. Interestingly, following the blocking of type I IFN signaling in diabetic mice, the lymphocytes exhibited restored proliferative capacity, decreased apoptosis, normal expression of PD-1, and normal phosphorylation of STAT1, STAT2, AKT and I**κ**B-**α**. Our data suggest that elevated levels of type I IFN during T1D trigger lymphocyte exhaustion and a defective lymphocyte-medicated immune response.

## 1. Introduction

Type 1 diabetes mellitus (T1D) is a chronic autoimmune disease caused by the specific destruction of pancreatic *β* cells, which produce insulin [1]. Extensive studies have focused on the role of the immune system in the development of T1D, from the initiation of disease to eventual beta-cell destruction [2, 3]. As both free-radical production and antioxidant defenses may be disturbed in diabetes [4], it has been suggested that oxidative stress may be partly responsible for the development of diabetic complications [5]. Consistent with this, oxidative stress has been implicated in the pathogenesis of insulin-dependent diabetes mellitus in several studies [6–8]. Increased levels of lipid peroxidation products and altered antioxidative enzyme activity were also reported in noninsulin-dependent diabetes mellitus [9].

In rodent models of diabetes, streptozotocin (STZ), a genotoxic methylating agent that is targeted to the *β* cells, is used to trigger the initial *β*-cell death leading to diabetes induction [10]. It has been documented that a single high dose of STZ (over 100 mg/kg body weight) causes extensive *β*-cell necrosis [11]. On contrary, either single moderate dose of STZ (60 mg/kg body weight) [12, 13] or multiple low doses for five consecutive days (40–50 mg/kg body weight) induce limited apoptosis, which elicits an autoimmune reaction that eliminates the remaining cells [10, 14, 15]. Moreover, rather than necrosis, apoptosis is the underlying mechanism of islet cell death, documented by findings that animals deficient in islet-associated caspase-3 are resistant to STZ effects [14]. 

Interferon-*α* (IFN-*α*) belongs to a group of pleiotropic cytokines in the type I family of IFNs [16]. Type I IFNs (IFN-*α*/*β*) influence the proliferation, differentiation, and function of numerous types of cells in the immune system [17]. IFN-*α* exerts broad but distinct effects on the innate and adaptive immune responses by signaling through a heterodimeric receptor composed of IFN-*α* receptor 1 (IFNAR1) and IFNAR2 [18]. Several studies have suggested that IFN-*α* is involved in the development of T1D; for example, higher levels of IFN-*α* mRNA and protein were detected in the pancreata of T1D patients than nondiabetic patients [19], and IFN-*α* treatment in patients with tumors or viral hepatitis is also associated with an increased incidence of T1D [20]. Additionally, overexpression of IFN-*α* in *β* cells was sufficient to induce T1D in nonautoimmune-prone C57BL/6 mice [21], whereas IFN regulatory factor 1-deficient NOD mice failed to develop insulitis and diabetes [22]. In addition, the proliferation of lymphocytes is reduced in diabetic rats [23], and the resultant decreased number of lymphocytes is likely a consequence of apoptosis [24]. However, certain studies have also shown that oral treatment in prediabetic NOD mice with IFN-*α* suppressed insulitis and diabetes [25]. Thus, the specific role of IFN-*α* in the disturbance of lymphocyte proliferation and function during T1D remains unresolved. Recently, our group has demonstrated that diabetic mice exhibited increased apoptosis, DNA fragmentation, chromatin condensation and cell shrinkage, prolonged elevation in IFN-*α* and TNF-*α* levels, and a clear reduction in spleen-homing T lymphocytes [26]. 

In the present study, we further investigated the relationship between type I IFN signaling and the survival/death and function of peripheral blood mononuclear cells (PBMCs) during T1D. To achieve this aim, we used a STZ-induced diabetic animal model lacking type I IFN signaling due to treatment with an anti-IFNAR1 blocking antibody.

## 2. Materials and Methods

### 2.1. Chemicals

Streptozotocin (STZ) was obtained from Sigma Chemicals Co. (St. Louis, MO, USA). The STZ was dissolved in cold 0.01 M citrate buffer (pH 4.50) and was always freshly prepared for immediate use (within 5 min).

### 2.2. Animals and Experimental Design

Laboratory Swiss Webster mice, weighing 25–30 g, were obtained from the Central Animal House of the Faculty of Medicine, Assiut University. All animal procedures were performed in accordance with the guidelines for the care and use of experimental animals set forth by the Committee for the Purpose of Control and Supervision of Experiments on Animals (CPCSEAs) and according to the protocol of the National Institute of Health (NIH). The animals were allowed to acclimate for 2 weeks before the experiment and were housed in metal cages in a well-ventilated room. These animals were maintained under standard laboratory conditions (25°C temperature, 60–70% relative humidity, and 12 hour light/dark cycle) and were fed a standard commercial pellet diet and water. Forty Swiss Webster mice were divided into three experimental groups: group 1, the nondiabetic control group (*n* = 10), was injected with the vehicle alone (0.01 M citrate buffer, pH 4.5); group 2, the diabetic group (*n* = 15), was rendered diabetic with an intraperitoneal injection of a single dose of STZ (60 mg/kg body weight) in 0.01 M citrate buffer (pH 4.5) [12, 13]; group 3 (*n* = 15) was rendered diabetic with the same injection but was also administered intraperitoneal injections with an anti-IFNAR1 antibody at a dose of 10 mg per kilogram of body weight daily for up to 20 days [27].

### 2.3. Monitoring the Immunosuppression in the Diabetic Animal Model

Control and STZ-induced diabetic mice were first immunized with a cell suspension of sheep red blood cells (SRBC; 5 × 10^8^ cells/mouse; i.p.) in PBS. Fifteen days after the first immunization, mice were then immunized with a second dose of SRBC (5 × 10^8^ cells/mouse; i.p.). Seven days after the second immunization the animals were bled and the sera were collected to evaluate the secondary humoral immune response by monitoring the levels of IgG and IgM. To measure mouse IgG and IgM, anti-SRBC serum samples were prepared at different dilutions in PBS-BSA 0*·*5%. Then, 10 mL of serum was incubated with 3 mL of 1% SRBC (PBS-BSA 0.5%) for 30 min at 4°C. The cells were washed three times and (PE) anti-IgG was added and incubated for 30 min at 4°C. Cells were washed and immunoglobulin was evaluated using flow cytometry analysis. Controls of SRBC incubated with labeled antibodies in the absence of serum were also carried out.

### 2.4. Histology, Immunohistochemistry, and Isolation of Pancreatic Islet *β* Cells

Paraffin-embedded pancreatic tissue was stained with hematoxylin and eosin (H&E) and examined by light microscopy. Pancreatic tissue was fixed overnight in a solution of freshly prepared 4% paraformaldehyde in 0.1 M PBS, pH 7.4, at 4°C. Samples were dehydrated and prepared as paraffin blocks and stained with H&E as mentioned above. For detection of insulin and CD3*ε* on sections, monoclonal anti-insulin, antiglucagon, and anti-CD3*ε* antibodies (1 : 100; DAKO), respectively, were used. The appropriate primary antibody was added in blocking buffer and incubated overnight at 4°C. Sections were washed and incubated in biotinylated secondary antibody at 1 : 2,000 for 2 h at room temperature, followed by washing and incubation with avidin biotin complex (Vectastain Elite ABC kit; Vector Laboratories, Burlingame, CA, USA) at 1 : 100 for 1 h at room temperature. Sections were counterstained with Mayer haematoxylin for 2 to 5 minutes and mounted. To isolate the islets, 2 mL of 2 mg/mL collagenase (type IV; Sigma) was injected into the common bile duct for pancreatic digestion. The digested pancreas was removed and incubated at 37° for 20 to 28 min. The digest was washed twice by resuspension in Hank's balanced salt solution (HBSS) and centrifuged at 400 ×g for 1 min at 4°C. The pellet was resuspended in HBSS and filtered through gauze. Islets were then handpicked in HBSS. Islet cells were maintained in suspension culture in RPMI 1640 supplemented with 10% fetal bovine serum, 100 U of penicillin/mL, and 100 *μ*g of streptomycin/mL and incubated at 37°C and 5% CO_2_ for 6 h before staining with Annexin V binding assay for apoptosis detection.

### 2.5. Blood Samples

At the end of the experiment, the mice were anesthetized with pentobarbital (60 mg per kilogram of body weight), the abdominal cavity was opened, and whole blood was drawn from the abdominal aorta. The plasma was obtained by low-speed centrifugation (1000 ×g for 20 minutes) and immediately stored at −80°C for subsequent cytokine profile analysis. PBMCs were also isolated using the Ficoll gradient method.

### 2.6. ROS Measurement

The ROS levels were determined using 2,7-dichlorodihydrofluorescein diacetate (H2DCF-DA) (Beyotime Institute of Biotechnology, Haimen, China). PBMCs (1 × 10^6^ cells) were directly treated with 10 mM H2DCF-DA dissolved in 1 mL 1X PBS at 37°C for 20 minutes. The fluorescence intensity was then monitored using an excitation wavelength of 488 nm and an emission wavelength of 530 nm.

### 2.7. Hydroperoxide Measurement

The hydroperoxide levels were evaluated using the Free Radical Analytical System (FRAS 2; Iram, Parma, Italy), a colorimetric assay based on the ability of hydroperoxide to generate free radicals after reacting with certain transitional metals.

### 2.8. Cytokine Measurement

The plasma cytokine profile (IL-1*α*, IL-1*β*, IL-6, and CXCL10) and IFN-*α* and IFN-*β* levels were determined by enzyme-linked immunosorbent assay (ELISA) using commercially available kits (R&D Systems, USA) according to the manufacturer's instructions. The cytokine concentrations were then calculated using a standard cytokine curve included on the same plate as the samples. 

### 2.9. Apoptosis Detection

Isolated PBMCs were fixed and permeabilized with 70% ice-cold ethanol for at least 1 hour and then washed twice in 1X PBS. DNA was stained by incubating the cells at 37°C for 1 hour in 40 *μ*g/mL propidium iodide and 100 *μ*g/mL DNase-free RNase in 1X PBS. Samples were analyzed by flow cytometry using a FACSCalibur flow cytometer (BD-Pharmingen). The FL2 red fluorescence channel was assessed on a linear scale, and the percentage of cells undergoing apoptosis was determined as the percentage of hypodiploid cells (sub G0/G1 peak). Dead cells were identified using the Trypan blue exclusion test.

### 2.10. Cell Proliferation Assay

To measure the proliferation and differentiation of lymphocytes, we performed a carboxyfluorescein diacetate succinimidyl ester (CFSE) assay. Typically, after cell division, CFSE labeling is distributed equally between daughter cells, each of which is therefore half as fluorescent as the parent cell. For this assay, after two washes in 1X PBS, the PBMCs from each group were resuspended at 5 × 10^6^ per milliliter of 1X PBS and stained with 0.63 *μ*M CFSE (Molecular Probes, Eugene, OR, USA) for 8 minutes at room temperature. The reaction was stopped with FBS, and the cells were washed three times in 1X PBS. The CFSE-labeled cells were then seeded onto 6-well plates with or without a mitogen cocktail (Molecular Probes) and grown for 4 days in cell culture medium. Data were collected for 50000 cells and analyzed using a BD FACSCalibur flow cytometer and FlowJo software (BD Biosciences). The proliferating lymphocyte subsets were distinguished by low CFSE staining. 

### 2.11. Programmed Cell Death 1 (PD-1) Protein Expression

Phycoerythrin (PE)-labeled anti-mouse PD-1 and isotype control IgG mAbs were purchased from BD Biosciences (San Jose, CA, USA). After washing with cold PBS, 1 × 10^6^ PBMCs from six animals per group were centrifuged at 300 ×g for 10 minutes at room temperature to pellet the cells. The cells were subsequently incubated with PE-labeled anti-mouse mAbs for 30 minutes at 4°C, followed by a final washing in cold 1X PBS. The cells were then washed twice and fixed in 1X PBS containing 2% paraformaldehyde. PD-1 expression on viable cells was then quantified by flow cytometry analysis.

### 2.12. Western Blot Analysis

Prior to Western blot analysis, 1 × 10^7^ PBMCs/mL from five animals per group were incubated in prewarmed RPMI-1640 without FCS and were either stimulated with 3000 IU/mL IFN-*α* for 5 minutes or left unstimulated. Lysates were then prepared as previously described [27], and equal amounts of the total cellular protein were subjected to SDS-PAGE and blotted onto a nitrocellulose membrane (Millipore, Bedford, MA, USA). After primary antibodies recognizing phospho-STAT1, STAT1, phospho-STAT2, STAT2, phospho-I*κ*B, I*κ*B, AKT, and phospho-AKT (Cell Signaling, UK) were diluted at 1 : 1000 in 1X TBS with 0.1% Tween-20 and 5% bovine serum albumin (BSA), the membrane was incubated overnight with a primary antibody on an orbital shaker at 4°C. Following the overnight incubation, the primary antibody was removed, and the membrane was washed three times in washing buffer for 5 minutes each. A horseradish peroxidase (HRP)-labeled goat anti-rabbit gamma globulin secondary antibody (Cell Signaling, UK) was diluted to 1 : 1000 and applied to the membrane for 1 hour at room temperature on an orbital shaker. The membrane was then washed three times in washing buffer for 5 minutes, followed by a single wash in distilled water for 5 minutes. The antigens were visualized using chemiluminescence (ECL, SuperSignal West Pico Chemiluminescent Substrate; Perbio, Bezons, France) and exposure to X-ray film (Amersham Biosciences, France). The ECL signal was specifically recorded on ECL Hyperfilm. To quantify the band intensities, the films were scanned, saved as TIFF files, and analyzed using NIH ImageJ software.

### 2.13. Statistical Analysis

The data were tested for normality using the Anderson-Darling test and for homogeneity variances prior to further statistical analysis. The data were normally distributed and are expressed as the mean ± standard error of the mean (SEM). Significant differences among groups were analyzed using a one- or two-way ANOVA followed by Bonferroni's test for multiple comparisons using PRISM statistical software (GraphPad Software). The data were also reanalyzed using a one- or two-way ANOVA followed by Tukey's posttest using SPSS software, version 17. Differences were considered statistically significant at *P* < 0.05.

## 3. Results

### 3.1. STZ-Induced Diabetic Mice Are Immunosuppressed

**Figure 1:**
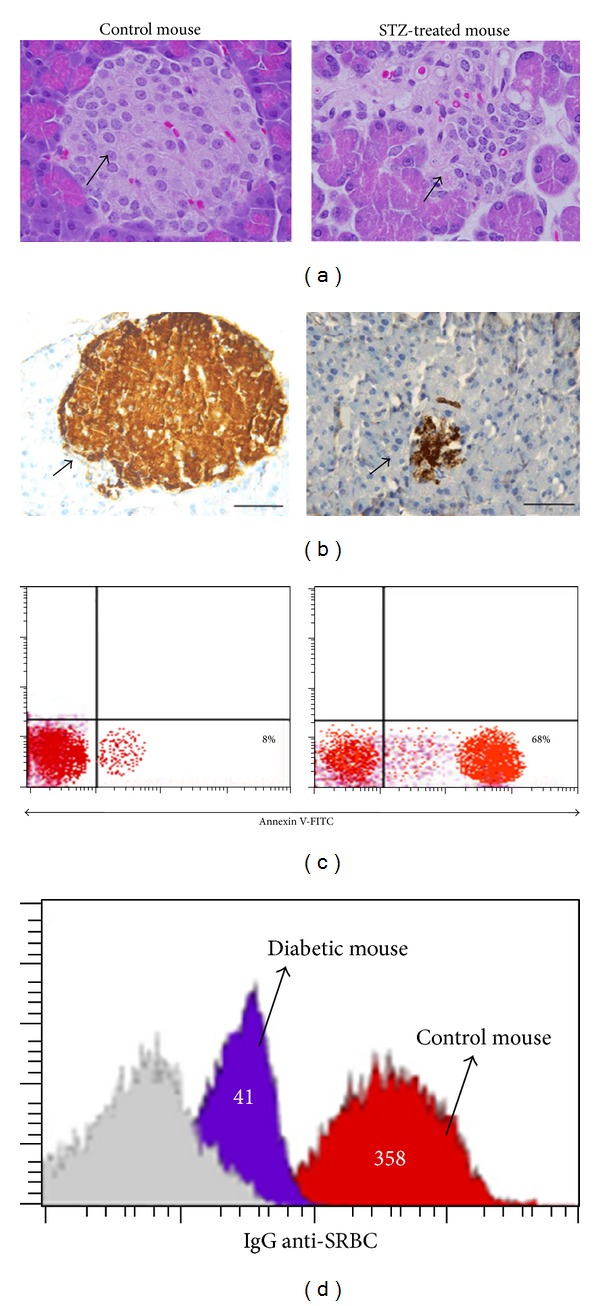
STZ-induced immunosuppressed diabetic mice. (a) Pancreatic sections of both control and STZ-treated mice were stained with H&E for light microscopy examination (magnification, ×40). (b) Pancreatic sections were immunohistochemically stained for the islet *β* cells (magnification, ×40). (c) Pancreatic islet *β* cells were isolated from control and diabetic mice, and the percentage of *β* cells that underwent apoptosis was monitored using Annexin V binding assay followed by flow cytometry analysis. (d) Control and diabetic mice were immunized with SRBC as described in the text. The level of IgG anti-SRBC level was compared between control and diabetic mice. Histograms show one representative experiment of three. Number in histograms represents the mean fluorescent intensity (MFI).

We first investigated the effect of STZ- (a single moderate dose of 60 mg/Kg of body weight) induced immunosuppression in the animal model that we used for further studies. First, pancreatic sections of both STZ-treated and naive mice were stained with H&E followed by examination using light microscopy. Data from one representative mouse out of three revealed a marked reduction in the pancreatic islets in STZ-treated animal as compared to naive mouse (Figure 1(a)). We then analyzed by immunohistology the architectures of insulin-production *β* cells within the pancreatic islets of STZ-treated and naive mice. As shown in Figure 1(b), result from one experiment out of three demonstrated that an obvious destruction of insulin-production *β* is STZ-treated mouse as compared to naive mouse. It has been documented that the dose of STZ that induces limited apoptosis of pancreatic *β* cells elicits an autoimmune reaction that eliminates the remaining *β* cells [10, 14, 15]. We therefore isolated the pancreatic *β* cells from naive and STZ-treated mice, and we then monitored the induction of apoptosis in the isolated *β* cells using Annexin V binding assay and flow cytometry analysis. We found that the percentage of *β* cells that underwent apoptosis was 68% in STZ-treated mouse versus 8% in control mice (Figure 1(c)). Furthermore, we have recently demonstrated that STZ-treated mice exhibited a marked and significant elevation in the apoptotic cells and subsequent reduction in the number of splenocytes as compared to naive mice, confirming that a single moderate dose of STZ (60 mg/Kg of body weight) induced immunosuppression [26]. Additionally, both naive and diabetic mice were immunized with sheep red blood cells (SRBC) as described in the text, and the level of IgG anti-SRBC was monitored as indicator of the humoral secondary immune response. As shown in Figure 1(d), the histograms of one representative experiment out of three demonstrated an obvious reduction in the levels of IgG anti-SRBC in STZ-treated (41) and naive mouse (358). Number in histograms represents the mean fluorescent intensity (MFI).

### 3.2. Blockade of Type I IFN Signaling in Diabetic Mice Decreases the Blood Levels of Oxidative Stress

To optimize the parameters and conditions of the animal models during experimentation, the blood glucose and insulin levels of the three groups of mice were monitored after diabetes induction (Table 1). The glucose levels in both the diabetic and type I IFN signaling-incompetent diabetic groups were significantly higher than those in the control group. In contrast, the insulin levels were significantly decreased in the diabetic and type I IFN signaling-incompetent diabetic groups compared to the nondiabetic control group. An examination of the plasma oxidative stress in the three groups revealed that the levels of ROS, hydroperoxide, and MDN were significantly increased in diabetic mice compared to nondiabetic control mice. Interestingly, blocking type I IFN signaling in diabetic mice significantly decreased the levels of ROS, hydroperoxide, and MDN. Diabetic mice exhibited an obvious and significant increase in the levels of IFN-*α* and IFN-*β* as compared to control nondiabetic mice. Blocking type I IFN receptor in diabetic mice partially and significantly restored the altered levels of IFN-*α* and IFN-*β* as compared to diabetic nontreated mice.

### 3.3. Plasma Cytokine Profile of Anti-IFNAR1-Treated Diabetic Mice

**Figure 2:**
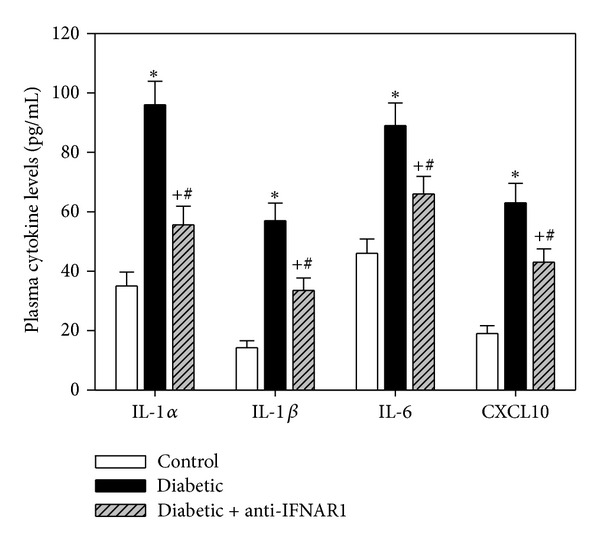
Plasma levels of proinflammatory cytokines are altered during diabetes. The levels of plasma cytokines (IL-1*α*, IL-1*β*, IL-6, and CXCL10) were measured in the three groups of mice using an ELISA. The results are presented as the picograms of cytokine per milliliter of plasma and are expressed as the mean ± SEM (*n* = 10). *P < 0.05 for diabetic versus control; ^#^ *P* < 0.05 for diabetic + anti-IFNAR1 versus diabetic; ^+^ *P* < 0.05 for diabetic + anti-IFNAR1 versus control.

We monitored the plasma levels of various proinflammatory cytokines that can alter immune cell function during diabetes in all groups of mice (Figure 2). The data acquired for five individual mice per group are shown. In the diabetic mice, we observed aberrant and significantly elevated levels of IL-1*α*, IL-1*β*, IL-6 and CXCL10 compared to the control group, which indicated prolonged proinflammatory conditions during diabetes. In contrast, the cytokine levels were significantly decreased in diabetic mice treated with anti-IFNAR1 compared to untreated diabetic mice. Thus, blocking type I IFN signaling during diabetes partially but significantly restored the levels of IL-1*α*, IL-1*β*, IL-6, and CXCL10.

### 3.4. In Vivo Blockade of IFNAR1 Restores PBMC Proliferation during Diabetes

**Figure 3:**
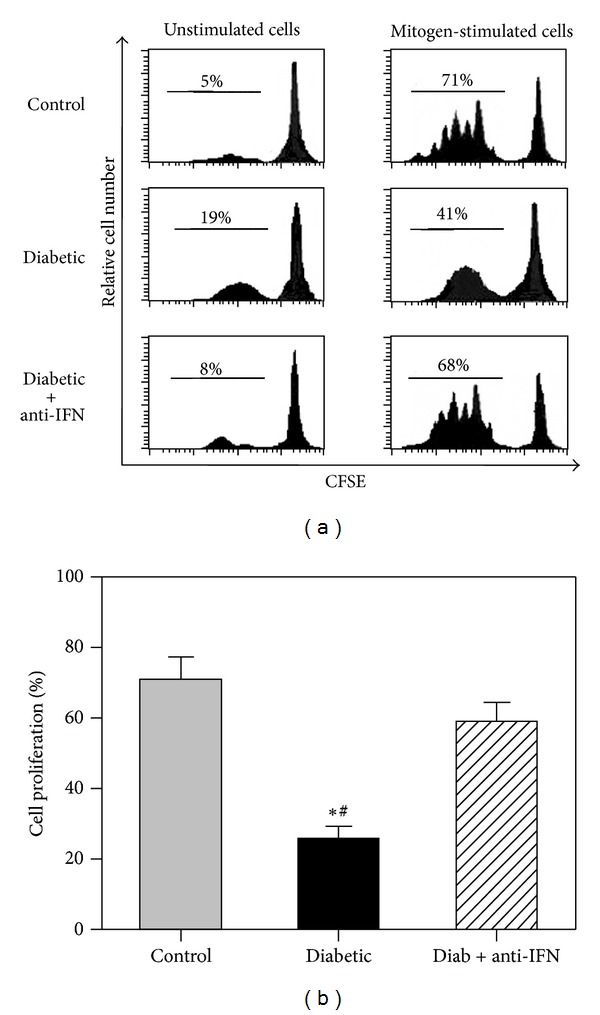
IFN-*α* decreases lymphocyte proliferation during diabetes. The CFSE fluorescence intensity for one representative experiment per group of mice was measured by flow cytometry, as shown in (a). The data acquired for five individual mice per group are shown in (b). To calculate the mitogen-induced specific proliferation of PBMCs, we subtracted the spontaneous proliferation (percentage of unstimulated cells in the absence of the mitogen) from the mitogen-induced proliferation (percentage of mitogen-stimulated cells). *P < 0.05 for diabetic versus control; ^#^ *P* < 0.05 for diabetic + anti-IFNAR1 versus diabetic; ^+^ *P* < 0.05 for diabetic + anti-IFNAR1 versus control.

 Our aim was to investigate whether the ability of PBMCs to proliferate in response to a mitogen, a phenomenon important for the maintenance and survival of immune cells, was altered in the diabetic group. In one representative experiment (Figure 3(a)), we found that the percentage of PBMCs that was spontaneously stimulated was markedly increased from 5% in the control group to 19% in the diabetic group and 8% in the anti-IFNAR1-treated diabetic group. When the cells were stimulated with the mitogen, the percentage of proliferating PBMCs was 71% in the control group versus 41% the diabetic group and 68% in the anti-IFNAR1-treated diabetic group. The data collected for five individual mice per group are shown in Figure 3(b). To calculate the mitogen-induced specific proliferation of the PBMCs, we subtracted the spontaneous proliferation (percentage of unstimulated cells in the absence of the mitogen) from the mitogen-induced proliferation (percentage of mitogen-stimulated cells), which revealed that the proliferative capacity of the PBMCs was significantly decreased from 72 ± 5.9% in the control group to 22 ± 2.1% in the diabetic group and 66 ± 4.9% in the anti-IFNAR1-treated diabetic group.

### 3.5. Blockade of IFNAR1 during Diabetes Reduces PBMC Apoptosis

**Figure 4:**
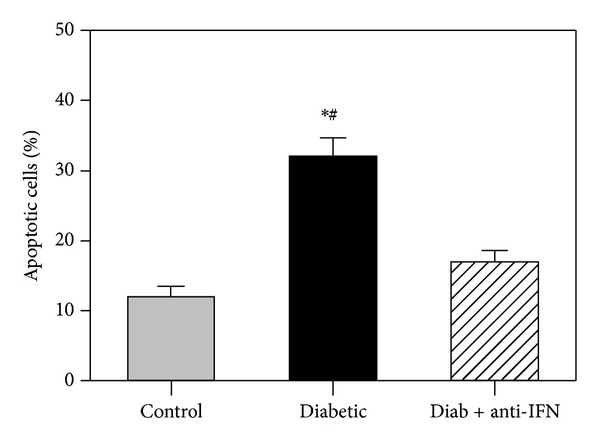
Increased lymphocyte apoptosis during diabetes. Isolated PBMCs were fixed and permeabilized, and the DNA was stained by incubating cells for 1 h in 40 *μ*L/mL propidium iodide and 100 *μ*L/mL DNase-free RNase in PBS. The samples were then analyzed by assessing the FL2 red fluorescence on a linear scale. The percentage of cells undergoing apoptosis was determined using flow cytometry. The results are expressed as the mean apoptotic cells ± SEM (*n* = 6). *P < 0.05 for diabetic versus control; ^#^ *P* < 0.05 for diabetic + anti-IFNAR1 versus diabetic; ^+^ *P* < 0.05 for diabetic + anti-IFNAR1 versus control.

To investigate the protective effect of blocking IFNAR1 on the survival of PBMCs during diabetes, isolated PBMCs were fixed and permeabilized by incubation in 70% ice-cold ethanol for at least 1 hour and were then washed twice in PBS. The DNA was stained by incubating the cells at 37°C for 1 hour with 40 *μ*L/mL propidium iodide and 100 *μ*L/mL DNAse-free RNAse in 1X PBS. The samples were then analyzed by assessing the FL2 red fluorescence on a linear scale. The percentage of cells undergoing apoptosis was determined using flow cytometry, and this was then used to determine the percentage of hypodiploid cells (sub-G0/G1 peak). The dead cells were identified using a Trypan blue dye exclusion test. As shown in Figure 4, the percentage of cells that underwent apoptosis was 12 ± 1.1 in the control group, and the percentage of apoptotic cells was significantly increased to 33 ± 3.1 in the diabetic group. In contrast, blocking type I IFN significantly reduced the percentage of apoptotic cells.

### 3.6. Blockade of IFNAR1 Partially Reduces PBMC Exhaustion during Diabetes

**Figure 5:**
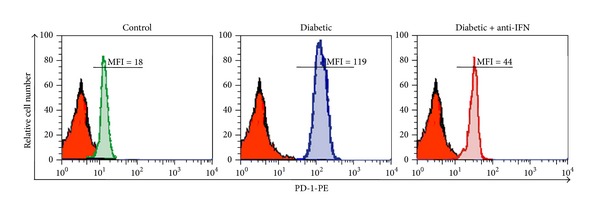
Increased PD-1 expression and lymphocyte exhaustion during diabetes. PBMCs were isolated from 5 mice (*n* = 5) per group and stained for 30 minutes at 4°C with PE-conjugated anti-PD-1 or isotype control IgG mAbs. The cells were then washed twice and fixed in PBS containing 2% paraformaldehyde, followed by flow cytometry analysis of PD-1 expression on viable cells. The numbers shown correspond to the mean fluorescence intensity (MFI) of PD-1 expression on labeled cells.

The data detailed in the previous section demonstrate that IFN-*α* induced aberrant and sustained activation, and therefore exhaustion, of lymphocytes during diabetes. To confirm this hypothesis, PD-1 expression was analyzed using flow cytometry (Figure 5). On the stained PBMCs, the MFI of PD-1 expression was low (18) on cells isolated from nondiabetic mice. In contrast, PBMCs isolated from the diabetic group were characterized by the aberrant upregulation of PD-1 expression (MFI = 119). Interestingly, cells isolated from the diabetic group with blocked type I IFN activity exhibited an obvious decrease in PD-1 expression (MFI = 44). As the upregulation of PD-1 expression is an indicator of continuous activation and exhaustion, the PBMCs of the diabetic group were likely exhausted, whereas blocking type I IFN signaling restored the functional state of the PBMCs.

### 3.7. Blockade of IFNAR1 Normalizes the Phosphorylation of AKT, I*κ*B-*α*, STAT1, and STAT2 during Diabetes

**Figure 6:**
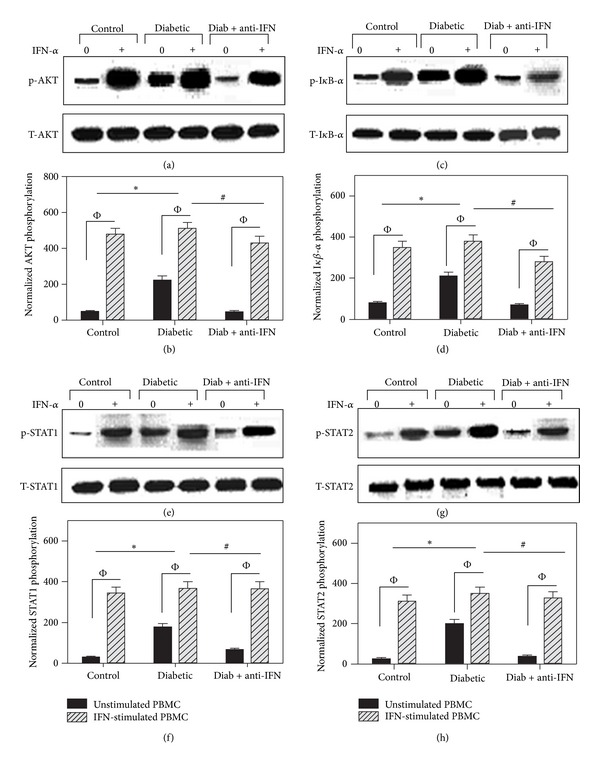
Lymphocytes from diabetic mice exhibit sustained phosphorylation of AKT, I*κ*B-*α*, STAT1, and STAT2 during diabetes. PBMCs were isolated from five mice per group (*n* = 5) and were either stimulated with IFN-*α* (2000 IU/mL) for 5 minutes or left unstimulated. Cell lysates were then prepared for Western blot analysis. The immunoblots from one representative experiment of five independent experiments are shown for the phosphorylation of AKT, I*κ*B-*α*, STAT1, and STAT2 (a, c, e, g). Total AKT, I*κ*B-*α*, STAT1, and STAT2 were used as equal loading controls. The data acquired for five individuals per group are expressed as the normalized average of the phosphorylated protein to the total relevant protein ± SEM, as shown in (b, d, f, h). *P* < 0.05 for unstimulated versus IFN-stimulated cells. The values for the level of normalized phosphoprotein in unstimulated cells were subtracted from those in IFN-stimulated cells to yield the specific phosphorylation values. The statistical analysis of the specific phosphorylation values revealed that *P < 0.05 for diabetic versus control; ^#^ *P* < 0.05 for diabetic + anti-IFNAR1 versus diabetic; ^+^ *P* < 0.05 for diabetic + anti-IFNAR1 versus control.

To explore the mechanisms by which IFN-*α* induced PBMC apoptosis, PBMCs were isolated from five mice per group, as described in the Materials and Methods section. The isolated cells were then either stimulated with IFN-*α* (2000 IU/mL) for 5 minutes or left unstimulated, and cell lysates were prepared for Western blot analysis. The immunoblots from one representative experiment of five independent experiments are shown (Figures 6(a), 6(c), 6(e), and 6(g)) for the phosphorylation of AKT, I*κ*B-*α*, STAT1, and STAT2. Total AKT, I*κ*B-*α*, STAT1, and STAT2 were used as equal loading controls. The data acquired from five individuals per group were analyzed using ImageJ software (France) and expressed as the normalized average of the phosphorylated protein to total relevant protein ± SEM (Figures 6(b), 6(d), 6(f), and 6(h)). Without IFN-*α* stimulation, the aberrant and sustained phosphorylation of AKT, I*κ*B-*α*, STAT1, and STAT2 was observed in the diabetic group as compared to the control and anti-IFNAR1-treated groups. These findings confirm that the PBMCs isolated from the diabetic group had been continuously stimulated with high levels of type I IFN *in vivo*. Moreover, IFN-*α* stimulation of PBMCs isolated from all groups of mice resulted in significant phosphorylation of AKT, I*κ*B-*α*, STAT1, and STAT2; however, if we subtracted the normalized phosphorylation of these proteins without stimulation from their values upon IFN-*α* stimulation, this calculation revealed that type I IFN signaling during diabetes was clearly perturbed. Interestingly, the diabetic group treated with anti-IFNAR1 exhibited normal signaling compared to the nondiabetic control group.

## 4. Discussion

Diabetes is widely believed to predispose patients to serious infections. However, the mechanisms linking diabetes with immunosuppression and defective lymphocyte function are not well defined. We previously demonstrated that type I IFN rescued B lymphocytes from apoptosis via PI3K*δ*/Akt, Rho-A, NF*κ*B, and Bcl-2/Bcl_XL_ [28]. We further extend this work and we revealed that blocking type I IFN signaling impairs antigen responsiveness of circulating lymphocytes and alters their homing to lymphoid organs [27]. Nevertheless, overproduction of type I IFN during diabetes could be a major factor of defective lymphocyte immune response. We therefore, in the present study, focused on investigating whether blocking type I IFN signaling during diabetes could restore the lymphocyte functions.

It has been shown that IFN-*α* is involved in the development of T1D, as higher levels of IFN-*α* mRNA and protein can be detected in the pancreata of T1D patients as compared to nondiabetic patients [19]. Furthermore, IFN-*α* may contribute to the initiation or acceleration of autoimmunity [20], and IFN-*α* is expressed in the islets of patients with newly diagnosed T1D [29]. In this context, we have recently demonstrated that blocking type I IFN signaling restored the number of lymphoid organ-homing lymphocytes in Swiss albino mice [27]. In the present study, we investigated whether blocking type I IFN signaling in a diabetic mouse model could improve lymphocyte function. Interestingly, the induction of diabetes in mice was associated with increased levels of glucose, oxidative stress and proinflammatory cytokines. One potential mediator of altered lymphocyte function is hyperglycemia, which can lead to enhanced oxidative stress, and play a key role in the development of defective immune responses during diabetes [30, 31]. ROS, hydroperoxide, and MDN are also thought to play a physiological role in oxidative stress and may cause damage to cellular components [32, 33].

In the current work, we examined the effect of the *in vivo* inhibition of type I IFN signaling on the survival/death and function of PBMCs (mainly lymphocytes) during T1D. It is generally accepted that proinflammatory cytokines are the major effectors of programmed cell death during the onset of type 1 diabetes mellitus [34]. Therefore, in the present study, the observed aberrant and significantly elevated levels of IL-1*α*, IL-1*β*, and IL-6 in diabetic mice indicated prolonged proinflammatory conditions. These results are consistent with previous studies revealing various alterations in the immune functions of diabetic patients. For example, diabetic patients have been reported to exhibit increased serum levels of proinflammatory cytokines, such as IL-6, which may play a significant role in the etiopathogenesis of insulin-dependent diabetes mellitus (IDDM) [35] and may increase inflammation and the development of vascular disease and atherosclerosis [36]. Elevated levels of IL-1 (IL-1*β*, IL-1*α*) also indicate a state of chronic inflammation in patients with IDDM [37]. Indeed, the results presented here demonstrate that blocking type I IFN signaling during diabetes partially yet significantly restored the levels of IL-1*α*, IL-1*β*, and IL-6. These findings are consistent with studies suggesting that IFN-*α* activates the peripheral immune system and induces the production of such proinflammatory cytokines as IL-1 and IL-6 during IDDM in humans [38].

We also noted elevated levels of CXCL10 (IP-10) in the diabetic mice compared to the control mice, further indicating prolonged proinflammatory conditions during diabetes. This observation is consistent with previous studies suggesting that HCV infection of pancreatic *β* cells may upregulate CXCL10 gene expression and protein secretion and recruit Th1 lymphocytes that secrete IFN-gamma and TNF-alpha, which in turn induces CXCL10 secretion by the pancreatic *β* cells and perpetuates the immune cascade that may lead to *β*-cell dysfunction [39, 40]. Moreover, a previous report revealed that CXCL10 induces cell death in cultured human pancreatic cells, leading to apoptosis and DNA fragmentation via CXCR3 signaling [41]. Interestingly, blocking type I IFN signaling during diabetes partially restored the levels of CXCL10. Our data are in agreement with reports that high IFN-*α* levels in the serum of systemic lupus erythematosus (SLE) patients induce the production of such proinflammatory chemokines as IP-10 [42]. Taken together, our results indicate that proinflammatory cytokines and chemokines are elevated during T1D in an IFN-*α*-dependent manner.

The present study also indicates that IFN-*α* induces the apoptosis of lymphocytes during T1D. The proliferative capacity of lymphocytes was significantly decreased in the diabetic group, whereas the percentage of apoptotic cells was significantly increased. However, blocking type I IFN signaling restored the proliferative capacity of lymphocytes and reduced the percentage of apoptotic cells. Thus, IFN-*α* may serve as a proapoptotic factor for lymphocytes during T1D. Consistent with our results, type I IFN has been reported to act as a proapoptotic factor in many cell types, such as lymphoma/leukemia cells [43]. Furthermore, we observed the upregulation of PD-1 expression on lymphocytes isolated from the diabetic group, which indicated that these cells had received continuous activation and subsequently became exhausted during T1D. Therefore, blocking type I IFN signaling was shown to restore PD-1 expression, whereas IFN-*α* signaling led to the continuous activation and exhaustion of lymphocytes. Our results are consistent with a previous report suggesting that PD-1 is likely critically involved in the development of peripheral tolerance as well as its loss during type 1 diabetes [44]. In the latter case, exhausted T cells lose the ability to combat invading pathogens, synthesize cytokines, and proliferate [45], and this lymphocyte exhaustion may be due to the sustained activation of lymphocytes during T1D.

Our data provide additional details regarding the IFN-*α*-signaling pathway during type I diabetes based on the use of antibodies to label proteins downstream of IFNAR1, such as STAT1, STAT2, AKT, and I*κ*B-*α*. These results indicate that IFN-*α* has a negative impact on the proliferation, survival, and differentiation of lymphocytes but enhances lymphocyte apoptosis via the spontaneous phosphorylation of the STAT1, STAT2, AKT and I*κ*B-*α* transcription factors. Aberrant activation of the key type I IFN intracellular signaling pathways in lymphocytes ultimately determines cell survival or death during T1D. Previous studies have indicated that STAT1 acts in a cell- and context-dependent manner and may lead to opposite outcomes, such as increased cell survival or apoptosis [46]. Furthermore, gene-knockout studies have shown that a deficiency in c-Rel in T cells leads to resistance to multiple low-dose streptozotocin-induced diabetes (MLDSD) [47]. The IFN signaling pathway leading to NF-*κ*B activation involves the tyrosine phosphorylation-dependent association of STAT3 with the IFN receptor, which results in the activation of a serine kinase cascade via PI-3K and AKT [48]. Our observation of the spontaneous phosphorylation of IFN-*α*-related signaling molecules indicates that aberrant AKT signaling may occur during T1D. Consistent with our results, the AKT signaling cascade is frequently deregulated in many types of cancer and, in some malignancies, has implications regarding tumor aggressiveness [49]. Taken together, our data highlighting the spontaneous phosphorylation of STAT1, STAT2, I-*κ*B, and AKT, mediated by IFN-*α*, in the diabetic group supports the continuous activation of lymphocytes during T1D and suggests that these cells have therefore lost the ability to proliferate and respond to invading pathogens. Furthermore, anti-IFNAR1 reduced PBMC apoptosis, and PBMC restored the cells' proliferation capacity and inhibited cellular exhaustion during T1D by impairing type I IFN signaling. However, further studies of the biological and immunological functions of different lymphocyte subpopulations including CD4+ T cell, CD8+ T cells, T-reg, NK, and naive and memory B cells in this diabetic model during blocking type I IFN signaling are underway.

## Figures and Tables

**Table 1:** Induction of diabetes mediates elevated levels of plasma oxidative stress. Blood biochemical parameters were measured in the three groups of mice, and the results are presented as the means ± SEM (*n* = 10), as described in Section 2.

Parameters	Control mice	Diabetic mice	Diabetic + anti-IFNAR1 mice
Glucose (mg/dl)	124 ± 9.3	332 ± 23*	301 ± 24^+^
Insulin (ng/mL)	5 ± 0.2	1.9 ± 0.1*	3.9 ± 0.35^+^
ROS (nmol/mL)	17 ± 3.6	65 ± 4.9*	29 ± 3.9^#+^
Hydroperoxide (mg/100 mL)	22 ± 4.7	54 ± 5.1*	34 ± 3.8^#+^
MDA (nmol/mL)	2.1 ± 0.6	8.1 ± 0.9*	4.1 ± 0.45^#+^
IFN-*α* (ng/mL)	1.722 ± 0.21	3.858 ± 0.586*	2.284 ± 0.264^#+^
IFN-*β* (ng/mL)	0.911 ± 0.19	2.97 ± 0.27*	1.72 ± 0.18^#+^

*P < 0.05 for diabetic versus control; ^#^ *P* < 0.05 for diabetic + anti-IFNAR1 versus diabetic; ^+^ *P* < 0.05 for diabetic + anti-IFNAR1 versus control.

## Abbreviations

IFN-*α*: Interferon-alpha
IL: Interleukin
I*κ*B-*α*: Nuclear factor of kappa light polypeptide gene enhancer in B-cell inhibitor, alpha
PKB/AKT: Protein kinase B
PD-1: Programmed cell death protein 1
STAT: Signal transducer and activator of transcription
T1D: Type 1 diabetes.
